# Astrocytic Response to Acutely- and Chronically-Implanted Microelectrode Arrays in the Marmoset (*Callithrix jacchus*) Brain

**DOI:** 10.3390/brainsci9020019

**Published:** 2019-01-23

**Authors:** Samuel A. Budoff, Kim M. Yano, Fernanda C. de Mesquita, Jhulimar G. Doerl, Maxwell B. de Santana, Manuela S. L. Nascimento, Ana Carolina B. Kunicki, Mariana F. P. de Araújo

**Affiliations:** 1 Edmond and Lily Safra International Institute of Neuroscience, Santos Dumont Institute, Macaíba, RN 59280-000, Brazil; samuel@edu.isd.org.br (S.A.B.); kim@edu.isd.org.br (K.M.Y.); fecmesquita@edu.isd.org.br (F.C.d.M.); jgdoerl@gmail.com (J.G.D.); manuela.mascimento@isd.org.br (M.S.L.N.); carol.bione@isd.org.br (A.C.B.K.); 2 Department of Physical Therapy, Federal University of Rio Grande do Norte, Natal, RN 59078-970, Brazil; 3 Brain Institute, Federal University of Rio Grande do Norte, Natal, RN 59056-450, Brazil; 4 Institute for Science and Technology of Water (ICTA), Federal University of Western Pará, Santarém, Pará 68040-255, Brazil; barbosadesantana@gmail.com

**Keywords:** gliosis, marmoset, microelectrode, astrocyte

## Abstract

Microelectrode implants are an important tool in neuroscience research and in developing brain–machine interfaces. Data from rodents have consistently shown that astrocytes are recruited to the area surrounding implants, forming a glial scar that increases electrode impedance and reduces chronic utility. However, studies in non-human primates are scarce, with none to date in marmosets. We used glial fibrillary acidic protein (GFAP) immunostaining to characterize the acute and chronic response of the marmoset brain to microelectrodes. By using densitometry, we showed that marmoset astrocytes surround brain implants and that a glial scar is formed over time, with significant increase in the chronic condition relative to the acute condition animal.

## 1. Introduction

Microelectrode implants are widely used in vivo to study the central nervous system (CNS) by recording neuronal activity or by stimulating specific brain regions. They are applied in brain–machine interfaces and neuromodulation devices [1]. Critically, the implants must be biocompatible, remaining stable in the CNS without causing cellular alterations that damage tissue or change electrode functionality [2,3,4,5,6].

Despite recent advances in the biocompatibility of implanted electrodes, tissue compatibility is still a concern. Astrocytes and microglia are cell populations known to be critical in the neuroinflammatory process [7,8]. They change their morphology to a hypertrophic conformation and secrete a wide range of soluble mediators when activated [9]. Upon recognizing an electrode as a foreign body, these cells will encapsulate it. The glial scar that is formed around the electrode acts as a barrier that empirically decreases electrophysiologic signal quality over time [2,3,10].

To date, most studies of neuroinflammation, due to the presence of microelectrodes, were performed in rodents [2,3,4,11,12]. Although rodents are the most widely used animal models in biomedical research, non-human primates (NHPs) are, phylogenetically, more similar to humans in neuroanatomical organization [13,14] and immunological responses [14,15].

Marmosets (*Callithrix jacchus*) are small NHPs that are increasingly being used in biomedical and preclinical research [16]. To date their innate neuroinflammatory response has not been characterized. It is critical that assumptions not be made when comparing immune responses between species, as interspecies differences, even with identical implantation procedures, have resulted in differences in glial scar formation [17,18].

In the present study, we analyzed the marmoset astrogliosis response to Teflon-coated tungsten microelectrode arrays for the first time, to our knowledge. Immunohistochemical analysis of glial fibrillary acidic protein (GFAP) was performed in brain tissue perfused three days (acute) and seven months (chronic) following implantation.

## 2. Materials and Methods

### 2.1. Animals

Two adult male common marmosets (*Callithrix jacchus*), weighing between 350 g and 450 g, were used in this study. Each subject was implanted with microelectrode arrays penetrating cortical and subcortical structures. The acute condition animal was perfused after 3 days, while the chronic animal was perfused 7 months after the implantation surgeries. Throughout the experiment, the animals were individually housed in cages (1.0 × 1.0 × 2.3 m) located in an outdoors vivarium with natural temperature and light cycle (~12/12h). They received 2 daily meals and water was available ad libitum. All procedures were carried out in strict compliance with the National Institute of Health Guide for the Care and Use of Laboratory Animals (NIH Publications No. 80-23) and were approved by the Ethics Committee for Animal Use of Alberto Santos Dumont Association for Research Support (protocols 08/2011 and 11/2011).

### 2.2. Microelectrode Arrays and Implant Surgery

The animals were implanted with two microelectrode arrays, one in each brain hemisphere. Each array had 32 Teflon-coated microelectrodes (diameter: 50 µm) arranged in bundles. Each bundle was composed of 4 to 6 microelectrodes, with an inter-microelectrode spacing of 300 µm. All surgical procedures were performed under aseptic conditions. Anesthesia was induced with ketamine hydrochloride (10 mg/kg. i.m.) and maintained with isoflurane (1.5%–5%; 1–1.5 L/min O_2_). The animals’ heads were shaved before they were placed in a stereotaxic apparatus. The skull was exposed and small craniotomies were made above the target brain areas. The dura mater was removed before each array was slowly inserted into the brain to minimize bleeding and tissue damage. Here we analyzed aggregate data from high quality microelectrode tracks, inserted into the motor cortex (M1) and putamen (Put) in the acute animal, and into the cingulate cortex (CC) in the chronic animal. The insertion coordinates were as follows: anteroposterior, mediolateral, and dorsoventral coordinates (mm) relative to interaural line [19]: M1 (10.0/6.5/14.4), Put (8.5/6.5/11.5), and CC (6.8/1.0/15.0). The arrays were anchored to screws attached to the skull via dental acrylic. Following the surgery, the animals were treated for 3 days with enrofloxacin (5 mg/kg; subcutaneous) and dexamethasone (0.5 mg/kg; intramuscular).

### 2.3. Immunohistochemistry

At the end of the survival times, the animals were intracardially perfused with 0.9% saline, followed by 4% paraformaldehyde, in 0.1 M phosphate buffer (pH 7.4). After perfusion, the brains were dehydrated, through sequential exposure to solutions of 20% and 30% sucrose, before being stored at −80 °C. The brains were cut into 50 μm coronal sections and placed on electrically-charged slides (SuperFrost Plus^®^, Fisher Scientific International, NH, USA). Immunohistochemical (IHC) detection of glial fibrillary acidic protein (GFAP) was performed using goat primary anti-GFAP, 1:500 (Santa Cruz Biotechnology, TX, USA), and biotinylated rabbit anti-goat secondary antibody, 1:200 (Vector Lab, CA, USA). After staining, the images were acquired with a CX9000 camera (MBF Bioscience Inc., Williston, VT, USA) coupled to an Axio Imager Z2 optical microscope (Zeiss, Berlin, Germany) using 2.5×, 20×, and 40× objectives.

### 2.4. Image and Statistical Analysis

IHC data were analyzed qualitatively and quantitatively. Qualitative analysis was performed to confirm the presence of morphologically typical astrocytes. GFAP-immunoreactive staining density, in 1-mm tissue adjacent to the tip of the microelectrode tracks, was assessed by optical densitometry using ImageJ (NIH http://rsb.info.nih.gov/ij/), following a protocol adapted from [11]. To control for differences in tissue staining and to gather relevant control data, we measured a 1 mm area in similar, non-implanted tissue from each section. Following measurement, average density from 50 μm segments were calculated and compared against the respective non-implanted tissue’s average data. Next, the non-implanted means were used as the respective denominator against the implanted means to control for section specific background noise, enabling relativistic comparisons between animals. No statistical difference was observed between the acute putamen and acute cortical regions, enabling relativistic comparisons between animals. Mann–Whitney U tests were performed following a Kolmogorov–Smirnov test that demonstrated the necessity of non-parametric analysis in this study. Comparisons using the non-parametric Mann–Whitney U test were judged to be statistically significant at *p* < 0.05.

## 3. Results

**Figure 1:**
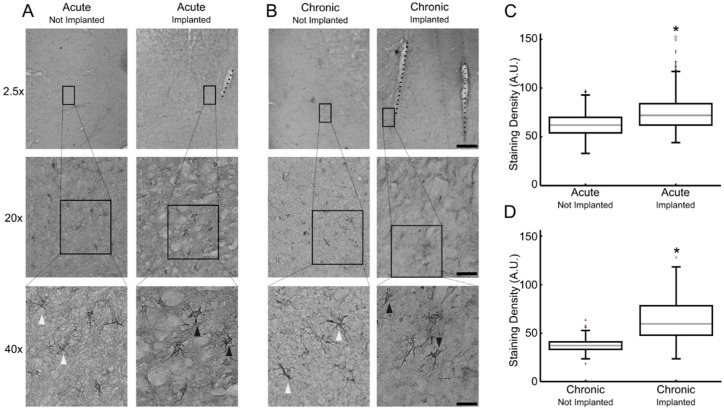
Astrocytes observed in brain tissue from marmosets with acute and chronic microelectrode implants. Representative images from glial fibrillary acidic protein (GFAP) staining in acutely- (**A**) and chronically-implanted (**B**) animals at 2.5×, 20×, and 40× objective magnifications. Representative inactive morphology astrocyte (white arrowhead), active morphology astrocyte (black arrowhead), and microelectrode tracks (black dashes) are highlighted. Scale bars indicate 350, 140, and 75 μm, for 2.5×, 20×, and 40× objectives, respectively. Box plots of the GFAP-immunostaining densities observed in acute (**C**) and chronic (**D**) sections; asterisks indicate statistically significant difference relative to respective not-implanted region *p* < 0.05. A.U.: Arbitrary units.

Morphologically typical astrocytes were observed in tissue at 20× and 40× objective magnifications following GFAP staining (Figure 1). The astrocytes in the non-implanted regions displayed inactivated morphology, with small cell bodies and long processes (Figure 1A,B, left panels). Astrogliosis was predominantly observed in the regions adjacent to the microelectrode tracks, in which stained cells presented hypertrophic cell bodies and thicker processes (Figure 1A,B, right panels). In addition, astrocyte activation was reflected by an increase in GFAP-immunoreactive staining density within 1 mm from the site of implantation; the median (Mdn) staining density was higher in the acute implanted (Mdn = 75.0) compared to the non-implanted region (Mdn = 62.5), U = 1172, *p* = 0.00, (Figure 1C), and in the chronic implanted (Mdn = 58.1) compared to the non-implanted region (Mdn = 38.0), U= 0.00, *p* = 0.00, (Figure 1D).

**Figure 2:**
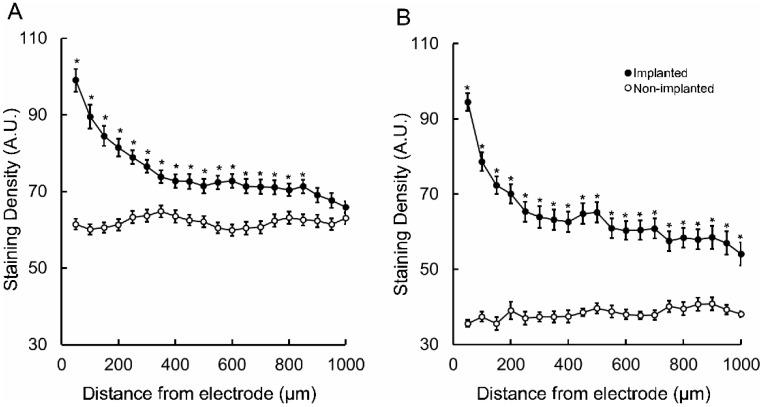
GFAP-immunostaining absolute density (mean ± SEM) within 1000 µm from the microelectrode tracks and within correspondent non-implanted regions. (**A**) acutely- and chronically-implanted (**B**) sections. Asterisks indicate statistically significant difference relative to respective not-implanted region *p* < 0.05. A.U.: arbitrary units.

**Figure 3:**
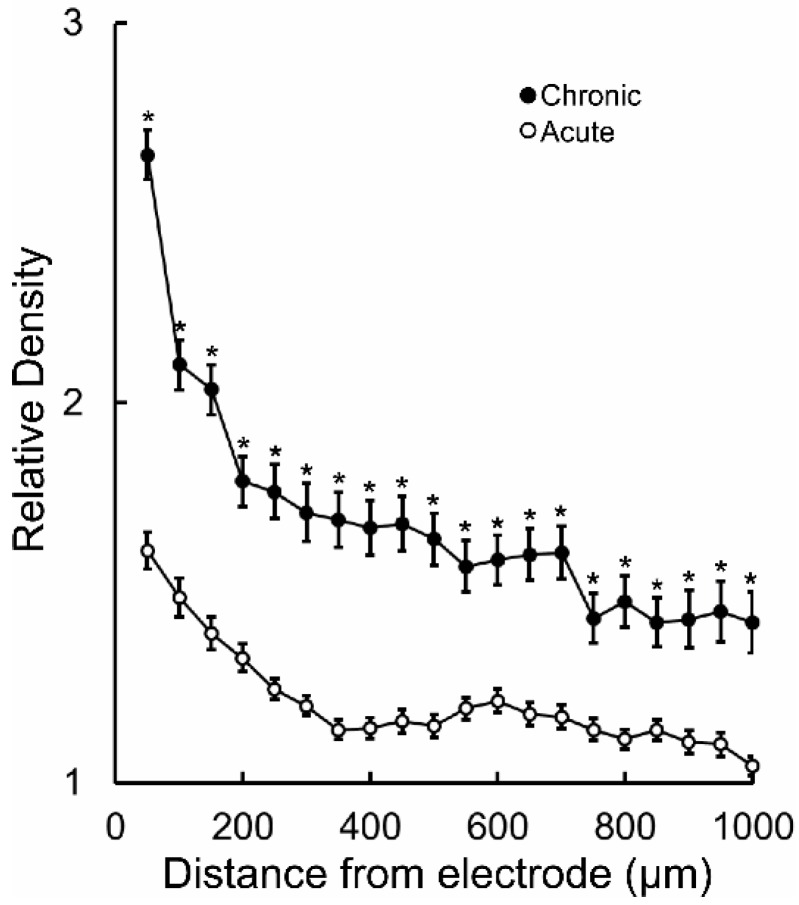
Normalized GFAP-immunostaining density (mean ± SEM) within 1000 µm from the microelectrode track in chronically- and acutely-implanted marmosets. Asterisks indicate statistically significant difference between acute vs. chronic regions of the same distance from microelectrodes implants *p* < 0.05.

Follow up comparisons of each 100 µm segment within the 1 mm area revealed that in the acute animal the increased GFAP-immunoreactive staining density was maintained until 850 µm from the microelectrode track (*p* < 0.05) (Figure 2A), whereas in the chronic tissue it was significantly elevated throughout the 1 mm area (*p* < 0.05) (Figure 2B). The absolute tissue staining was, on average, higher in the acute tissue, when parsing the data by distance. This is not an atypical outcome in IHC experiments. Thus, it was critical that normalization be performed before comparing acute to chronic GFAP-immunoreactive staining density. Following normalization against contralateral control tissue, statistical testing of each point separated by distance revealed that relative astrocyte density was significantly increased in the region adjacent to the chronic implants throughout the 1 mm area studied (*p* < 0.05) (Figure 3).

## 4. Discussion

Here we studied, for the first time, to our knowledge, the astrogliotic response of healthy marmosets to implanted microelectrodes. Previously, the only other work that has studied GFAP-immunoreactivity in marmosets was in animals with chemically induced epilepsy [20]. We observed that astrocytes proximal to implanted microelectrodes have the characteristic active morphology described in other species. Furthermore, we observed that as distance from the microelectrode increases, the GFAP-immunoreactive staining density drops toward non-implanted levels. These observations are similar to those made in rodents [5,10,18]. However, we observed an elevation above baseline that extended to at least 1000 µm in the chronically-implanted tissue, while GFAP IHC in rodents tends to return to baseline before 700 µm [5,10,18]. This discrepancy may be due to differences in electrode design, surgical procedures, or implantation time. Another possibility is that such discrepancies are related to interspecies differences. Relevantly, in rhesus macaques reactive astrocytes were found up to 1 cm away from the electrode tracks [21], highlighting an important difference between the rodent and primate immune responses.

In addition, we observed that the GFAP-immunoreactive staining density was higher in the tissue surrounding chronic implants compared to acute implants. This densitometric result agrees with our hypothesis that a glial scar is increasingly formed over time in marmosets. This result also agrees with evidence from other mammals that the glial scar will become more compressed and; therefore, darker in its immunoreactivity, closer to the electrode over time [5,10,18,21,22,23,24,25].

The present work is important because it brings to bear histologic data confirming marmoset astrocytes respond to implants by forming a glial scar. Though this study is limited by the comparison of an individual animal in each condition, we expect future work will similarly reinforce the differences in astrocytic activation noted between primates and rodents. Most relevant to the neuroscientific community at large, our group has previously described a decrease in the signal quality of microelectrodes chronically implanted in marmosets [22]. Thus, it is likely that astrocytes are, at least partly, responsible for the previously observed diminished electrophysiologic signal quality over time.

## 5. Conclusions

The findings of this study show that astrocytic response to foreign implants in the marmoset brain is similar to the response found in other mammals, including humans. Future studies in marmosets will further untangle the mechanisms governing astrogliosis, including the role of marmoset microglia. We hope that this work will inspire more detailed evaluations of the interspecies differences in how glial cell types maintain homeostasis. Importantly, future research can further build on this work with the potential to improve biocompatibility of neural implants via pharmaceutical or material science innovations.
